# Acupuncture combined Bobath approach for limbs paralysis after hypertensive intracerebral hemorrhage: A protocol for a systematic review

**DOI:** 10.1097/MD.0000000000014750

**Published:** 2019-03-08

**Authors:** Guang-fu Song, Xin Li, Yao Feng, Chang-hong Yu, Xiao-yu Lian

**Affiliations:** a Department of Neurosurgery; b Department of Neurology; c Department of Chinese Medicine; d Department of Gastroenterology, First Affiliated Hospital of Jiamusi University, Jiamusi, China.

**Keywords:** acupuncture, Bobath approach, effectiveness, hypertension, intracerebral hemorrhage, limbs paralysis, safety, systematic review

## Abstract

**Background:**

Previous studies have reported that acupuncture combined Bobath approach (BA) can be used to treat limbs paralysis (LP) after hypertensive intracerebral hemorrhage (HICH) effectively. However, no systematic review has explored its effectiveness and safety for LP following HICH. In this systematic review, we aim to assess the effectiveness and safety of acupuncture plus BA for the treatment of LP following HICH.

**Methods:**

The following 7 databases will be searched from their inception to the February 1, 2019: Cochrane Central Register of Controlled Trials, EMBASE, PUBMED, the Cumulative Index to Nursing and Allied Health Literature, the Allied and Complementary Medicine Database, Chinese Biomedical Literature Database, and China National Knowledge Infrastructure without any language restrictions. The randomized controlled trials (RCTs) of acupuncture plus BA that evaluate the effectiveness and safety for LP after HICH will be included. The methodological quality of all included studies will be assessed by using Cochrane risk of bias tool. Two authors will independently perform study selection, data extraction, and methodological quality evaluation. Any disagreements occurred between 2 authors will be resolved by a third author involved through discussion. Data will be pooled and analyzed by using RevMan 5.3 Software.

**Results:**

This review will evaluate the effectiveness and safety of acupuncture combined BA for LP following HICH. The primary outcome is limbs function. The secondary outcomes are muscle strength, muscle tone, and quality of life, as well as the adverse events.

**Conclusion:**

The results of this study will summarize the latest evidence of acupuncture combined BA for LP following HICH.

## 1 Introduction

Hypertensive intracerebral hemorrhage (HICH) is a devastating and costly disorder, which often accompanies high mortality and morbidity.^[1–4]^ Many factors are reported to account for this condition, such as hypertension, current smoking, excessive alcohol consumption, hypocholesterolemia, and any other factors, especially for the hypertension.^[5–8]^ It has been reported that HICH account for 10% to 20% of all strokes.^[9,10]^ Moreover, its incidence still increases with advanced age.^[11]^ Patients experience such disorder often manifest with headache, nausea, and vomiting initially, then followed by the limbs paralysis (LP), decreased consciousness, difficulty speaking, or sensitivity deficits and so on.^[12,13]^ Of these, LP is one of the trickiest conditions, and greatly affects the quality of life in patients with such disorder.^[14–16]^

Acupuncture and Bobath approach (BA) are both reported to widely treat LP after HICH, and also have achieved very satisfied outcome results.^[17–23]^ However, no systematic review has evaluated its effectiveness and safety with higher level evidence. Thus, it is very necessary to conduct a systematic review and meta-analysis to assess the effectiveness and safety of acupuncture combined with BA for the treatment of LP following HICH.

## 2 Methods and analysis

### 2.1 Eligibility criteria

#### 2.1.1 Participants/population

Patients with LP after HICH, regarding sex, age, and race will be considered for inclusion. However, if LP is diagnosed before the HICH, or caused by other disorders will not be included.

#### 2.1.2 Interventions/exposure

Any types of acupuncture combined with BA alone will be utilized to treat LP in the experimental group. The treatments in the control group can be any kinds of interventions, except the acupuncture, BA or combination of both.

#### 2.1.3 Study types

We will include randomized controlled trials (RCTs) of acupuncture combined with BA for the treatment of HICH without any restrictions. Studies will be excluded if they are nonclinical trials, case studies, crossover studies, non-RCTs, and quasi-RCTs.

#### 2.1.4 Outcome measurements

The primary outcome includes limbs function, as measured by the Fugl-Meyer Assessment scale, or other associated scales. The secondary outcomes include muscle strength, as assessed by the motricity index or other related scored tools; muscle tone, as evaluated by modified Ashworth scale, or other relevant scales; and quality of life, as examined by activities of daily living scale or any other specific scales. In addition, adverse events are also assessed.

### 2.2 Literature search

We will search the following 7 databases from their inception to the February 1, 2019: Cochrane Central Register of Controlled Trials (CENTRAL), EMBASE, PUBMED, the Cumulative Index to Nursing and Allied Health Literature, the Allied and Complementary Medicine Database, Chinese Biomedical Literature Database, and China National Knowledge Infrastructure without any language restrictions. The studies of RCTs regarding the effectiveness and safety of acupuncture plus BA for LP after HICH will be included. The detailed strategy of CENTRAL is presented in Table 1. The equivalent search strategies will be applied to other databases.

**Table 1:**
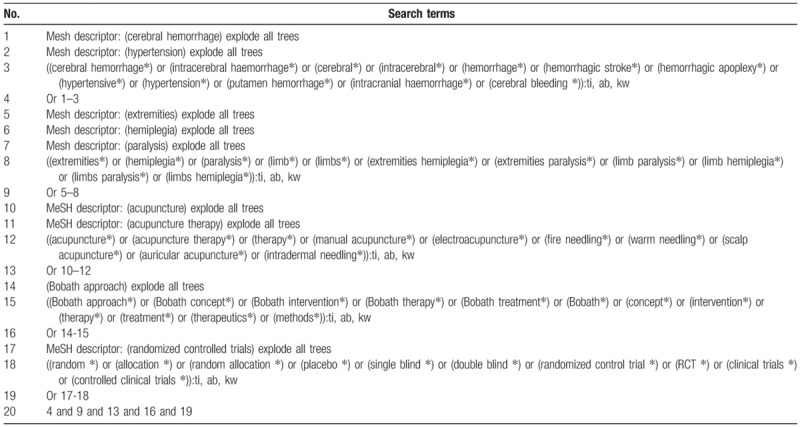
Search strategy applied in CENTRAL database.

The secondary data sources include Google scholar, website of clinical registrations, reference lists of all relevant reviews and included studies, as wells as the conference proceedings.

### 2.3 Data selection

The NoteExpress 3.2.0 will be used for study selection. Two authors will independently scrutinize the titles or abstracts initially, and then full texts will be read for eligible trials based on the predefined eligibility criteria. All study selection procedures will present in flowchart and will follow the Preferred Reporting Items for Systematic review and Meta-Analysis Flow Chart. The flowchart will include exact reason of inclusion or exclusion for each study at each stage. Any disagreements about the study selection will be settled down through discussion with a third author.

### 2.4 Data extraction and management

Two authors will extract following information from each included study and will save all the data in a data extraction sheet: general information (first author, published year, region, age, sex, ethnicity, disease types); relevant study methods (sample size, randomization, allocation, and blinding); interventions methods (details of interventions, including dosage, frequency, treatment duration); and outcome measurements (primary and secondary outcomes, adverse events, and any others). Any divergences between 2 authors will be resolved by a consensus or arbitration with a third author.

### 2.5 Dealing with missing data

The primary authors will be contacted to acquiring the insufficient or missing data if any of them arise. If we are not able to obtain those data, we will just analyze the available data, and also will discuss its potential impact in the discussion section.

### 2.6 Risk of bias assessment

Two independent authors will assess the methodology quality for each included study by using Cochrane risk of bias tool. This tool comprises 7 domains, and each one will classify into 3 levels: low, unclear, and high risk of bias. A third author will be invited to tackle the oppositions between 2 authors.

### 2.7 Reporting bias

We will also plan to conduct funnel plot and Egg's regression to detect the reporting bias if >10 eligible studies are included.

### 2.8 Statistical analysis

The statistical analysis will be carried out by using RevMan 5.3 software. Continuous data will be synthesized and shown as mean difference or standardized mean difference with 95% confidence intervals (CIs), while the dichotomous data will be synthesized and presented as risk ratio with 95% CIs.

Heterogeneity among included studies will be identified by using *I*^2^ test. *I*^2^ ≤50% is regarded as having fair heterogeneity, and fixed-effect model will be used to pool the data. Otherwise, heterogeneity is considered as mild or significant and random-effect model will be applied to pool the data. Meanwhile, subgroup analysis is suggested to be performed according to the different types of treatments, control interventions, and outcome measurements. If the heterogeneity remains substantial after subgroup analysis, then data will not be pooled, and a narrative summary will be presented instead. Sensitivity analysis will be carried out to ensure the robustness of pooled results by removing low quality studies.

## 3 Discussion

This systematic review will be conducted to evaluate the effectiveness and safety of acupuncture combined with BA for the treatment of LP in patients with HICH. To our best knowledge, no previous systematic review has addressed this issue. Thus, this systematic review will first assess the effectiveness and safety of acupuncture plus BA for treating LP following HICH.

In this systematic review, we will search as comprehensive data sources as possible without any language restrictions. All potential studies regarding the acupuncture plus BA for the treatment of HICH will be fully considered. The results of this systematic review may provide an up-to-date summary of the current evidence on the effectiveness and safety of acupuncture plus BA for LP following HICH.

## Author contributions

**Conceptualization:** Guang-fu Song, Yao Feng, Chang-hong Yu.

**Data curation:** Guang-fu Song, Xin Li, Xiao-yu Lian.

**Formal analysis:** Guang-fu Song, Xin Li, Yao Feng, Chang-hong Yu.

**Funding acquisition:** Guang-fu Song.

**Investigation:** Xiao-yu Lian.

**Methodology:** Guang-fu Song, Xin Li, Yao Feng, Chang-hong Yu.

**Project administration:** Xiao-yu Lian.

**Resources:** Guang-fu Song, Xin Li, Yao Feng, Chang-hong Yu, Xiao-yu Lian.

**Software:** Guang-fu Song, Xin Li, Yao Feng.

**Supervision:** Xiao-yu Lian.

**Validation:** Xin Li, Yao Feng, Chang-hong Yu, Xiao-yu Lian.

**Visualization:** Xin Li, Chang-hong Yu.

**Writing – original draft:** Guang-fu Song, Xin Li, Xiao-yu Lian.

**Writing – review and editing:** Guang-fu Song, Xin Li, Yao Feng, Chang-hong Yu, Xiao-yu Lian.
